# Trend of Outcome Metrics in Recent Out-of-Hospital-Cardiac-Arrest Research: A Narrative Review of Clinical Trials

**DOI:** 10.3390/jcm12227196

**Published:** 2023-11-20

**Authors:** Natalie N. Htet, Daniel Jafari, Jennifer A. Walker, Ali Pourmand, Anna Shaw, Khai Dinh, Quincy K. Tran

**Affiliations:** 1 Department of Emergency Medicine, Stanford University, Stanford, CA 94305, USA; nhtet@stanford.edu; 2 Donald and Barbara Zucker School of Medicine Hofstra Northwell, Hempstead, NY 11549, USA; djafari@northwell.edu; 3 Department of Emergency Medicine, North Shore University Hospital, Manhasset, NY 11030, USA; 4 Department of Emergency Medicine, Baylor Scott and White All Saints Medical Center, Fort Worth, TX 76104, USA; jennifer.walker@bswhealth.org; 5 Department of Emergency Medicine, Burnett School of Medicine, Texas Christian University, Fort Worth, TX 76109, USA; 6 Department of Emergency Medicine, George Washington University School of Medicine and Health Sciences, Washington, DC 20037, USA; pourmand@gwu.edu; 7 Research Associate Program in Emergency Medicine and Critical Care, Department of Emergency Medicine, University of Maryland School of Medicine, Baltimore, MD 21201, USA; 8 Department of Emergency Medicine, University of Maryland School of Medicine, Baltimore, MD 21201, USA; 9 Program in Trauma, The R Adams Cowley Shock Trauma Center, University of Maryland School of Medicine, Baltimore, MD 21201, USA

**Keywords:** cardiac arrest, outcome, intervention, patient-related outcome

## Abstract

Cardiopulmonary resuscitation (CPR) research traditionally focuses on survival. In 2018, the International Liaison Committee on Resuscitation (ILCOR) proposed more patient-centered outcomes. Our narrative review assessed clinical trials after 2018 to identify the trends of outcome metrics in the field OHCA research. We performed a search of the PubMed database from 1 January 2019 to 22 September 2023. Prospective clinical trials involving adult humans were eligible. Studies that did not report any patient-related outcomes or were not available in full-text or English language were excluded. The articles were assessed for demographic information and primary and secondary outcomes. We included 89 studies for analysis. For the primary outcome, 31 (35%) studies assessed neurocognitive functions, and 27 (30%) used survival. For secondary outcomes, neurocognitive function was present in 20 (22%) studies, and survival was present in 10 (11%) studies. Twenty-six (29%) studies used both survival and neurocognitive function. Since the publication of the COSCA guidelines in 2018, there has been an increased focus on neurologic outcomes. Although survival outcomes are used frequently, we observed a trend toward fewer studies with ROSC as a primary outcome. There were no quality-of-life assessments, suggesting a need for more studies with patient-centered outcomes that can inform the guidelines for cardiac-arrest management.

## 1. Introduction

The number of trials in the field of out-of-hospital cardiac arrest (OHCA) has grown exponentially throughout the last decade, largely with a focus on increased survival as a key metric for the effectiveness of interventions [1]. In 2018, in an effort to clarify meaningful outcomes for ongoing research, the International Liaison Committee on Resuscitation published the Core Outcome Set for Cardiac Arrest (COSCA) in Adults [2]. The COSCA initiative process painstakingly reviewed the literature for outcome data, created a priority list that was based on clinicians’, patients’, and their relatives/partners’ preferences, and derived an outcome set based on the consensus of an international advisory panel.

The literature review utilized for the COSCA process [3] affirmed that, within cardiopulmonary resuscitation research, there was a large variation in outcome metrics, such as the types of outcomes, the timing of when to measure outcomes, and the methods. For example, in the 61 included randomized controlled trials, survival was the most reported outcome (85.2%); however, there were 39 individual ways to assess this outcome. Furthermore, many outcomes (41%) were physiologic variables related to body structure or body function, such as heart rate or biomarkers. While the methods of measurement of physiologic data points were also heterogeneous, these outcomes are likely less relevant to patient-centered outcomes. Notably, none of the studies included health-related quality-of-life measurements.

After the outcome data were extracted from the COSCA systematic review, surveys were completed by clinicians, patients, and their relatives [4,5,6]. Importantly, patients and partners consistently ranked life-impact outcomes at 1 year, including emotional well-being and family impact, as important [5]. This is largely consistent with other studies on post-intensive-care syndrome (PICS), demonstrating that outcomes after surviving critical illness, including neurocognitive injury, physical debility, and psychosocial impact, are all patient-centered metrics that have historically been of little focus and poorly understood, yet have wide implications [7,8]. A recent study did look at out-of-hospital-cardiac-arrest survivors and the incidence of PICS at 3- and 12-month follow-up [9]. That study found that 50% of survivors experienced PICS at 3-months and 47% at 12-month follow-up [9].

Based on the systematic review, survey results, and panel discussion, the COSCA advisory group recommended that researchers include several core outcomes in ongoing cardiopulmonary resuscitation research [2]. These outcomes focus on three domains: survival, neuroprognostication, and health-related quality of life. Specifically, the panel recommended measuring (a) survival at hospital discharge, at 30 days, or both; (b) neurologic function measured by mRS at hospital discharge, at 30 days, or both; and (c)-health-related quality of life measured with least one tool at 90 days and at intervals up to 1 year after cardiac arrest. They recommended using the Health Utilities Index (HUI3), the Short-Form 36-Item (SF-36v2) Health Survey, and the EuroQol 5D-5L (EQ-5D-5L) as tools to determine this outcome of quality of life. 

Intuitively, the concepts of cardiopulmonary resuscitation outcomes do overlap. The return of spontaneous circulation, for example, is necessary for calculating more distant neurologic outcomes, even at 30 days [10]. Similarly, quality-of-life metrics are dependent on neurologic recovery. The return of spontaneous circulation as a primary outcome, however, is not necessarily a valuable, patient-centered outcome. When developing large trials and publishing association guidelines, it is important to focus on patient-centered outcomes that are consistently measured and meaningful. The COSCA outcome set provided that framework.

This narrative review aims to search the published literature since the publication of the COSCA outcomes in 2018 to determine the trend of outcome metrics that have been measured and to compare whether these outcomes align with the COSCA recommendations.

## 2. Methods

### 2.1. Study Selection

The PubMed database was searched from 1 January 2019 to 22 September 2023, using the search terms “(intervention) AND (“Out-of-Hospital Cardiac Arrest” [Mesh] OR “Heart Arrest” [Mesh])”. We included studies starting in January 2019, rather than in the COSCA publication year of 2018, in order to increase the likelihood that researchers would have time to incorporate additional patient-centered outcomes as recommended by the COSCA guidelines into their research methods. Our inclusion criteria were randomized controlled trials, prospective observational trials, or secondary analyses of prospective observational studies in adult human subjects that evaluated any diagnostic or therapeutic interventions in out-of-hospital cardiac arrest and reported any patient-related outcomes. We excluded studies that did not report any patient-related outcomes, such as studies assessing levels of biomarkers, non-original publications (reviews, meta-analyses), and conference proceedings. Studies not available in full-text English language were excluded. Two investigators independently screened the titles and abstracts for eligibility, and a third investigator adjudicated any discrepancies. All studies required agreement from at least two investigators to be included in the analysis. This review did not involve any human subjects; thus, it was not submitted to the Institutional Review Board at the Principal Investigator’s institution.

### 2.2. Data Collection

The data for the assessments included the demographic information (year of publication, country of study, study design, sample size) of each article and the patient-related primary outcomes and secondary outcomes (survival, neurofunctional outcomes, quality of life). In the first trial for data collection, the interrater agreement between investigators was 96%, so our standardized datasheet was well designed, and the data were validated.

## 3. Results

Our search identified 219 results, and after screening, we included 89 studies for analysis (Appendix A). There were 42 (47%) randomized trials, 37 (42%) second analyses of previous randomized trials, and 10 (11%) observational studies (Table A1).

For the primary outcome, 31 (35%) studies used an assessment for neurocognitive functions, while 27 (30%) used survival as their outcome. There were 8 (9%) studies using any neurocognitive assessment at hospital discharge, and most studies (22, 25%) assessed neurocognitive function beyond 30 days. In terms of survival as a primary outcome, four (4%) and seven (8%) studies used survival to hospital admission and hospital discharge, respectively. There were 4 (5%) and 12 (13%) studies that assessed survival at 30 days and beyond 30 days, respectively (Table A1).

**Figure 1:**
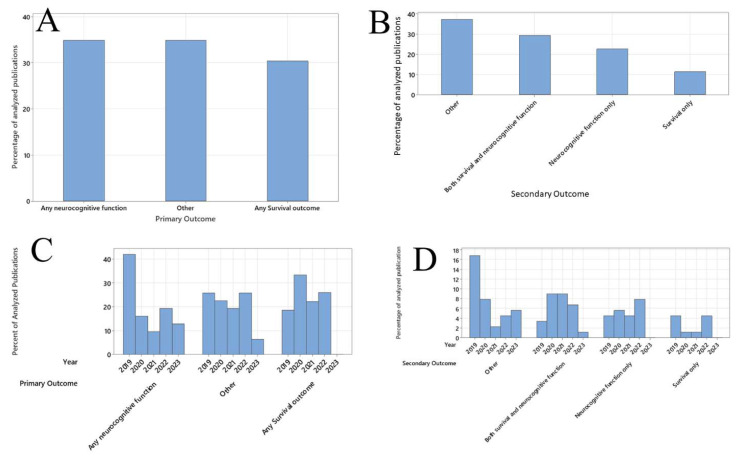
Trend of outcomes among publications involving patients with out-of-hospital cardiac arrest being included in this review. (**A**) Percentages of different categories of primary outcome, among the analyzed publications. (**B**) Percentages of different categories of all secondary outcomes, among the analyzed publications. (**C**) Percentages of different categories of primary outcome, in each year from 2019 to 2023. (**D**) Percentages of different categories of secondary outcome, in each year from 2019 to 2023.

For secondary outcomes, neurocognitive function, at any time of assessment, was present in 20 (22%) studies, and survival at any time of assessment was used as the secondary outcome in 10 (11%) studies. Twenty-six (29%) studies used both survival and neurocognitive function. Thirty-one (35%) studies listed other primary outcomes outside of the COSCA guidelines (Figure 1A).

Among all the studies, there were higher percentages of studies being published in 2019 that used neurocognitive functions as a primary outcome. The percentages of studies that used survival at any time period as a primary outcome appeared to be unchanged between 2019 and now (Figure 1C). On the other hand, the number of studies that used both neurocognitive function and survival or the number of studies that used only neurocognitive function as a secondary outcome remained the same since 2019. The number of studies that reported only survival as their secondary outcome was decreasing in 2021–2022 (Figure 1D).

**Figure 2:**
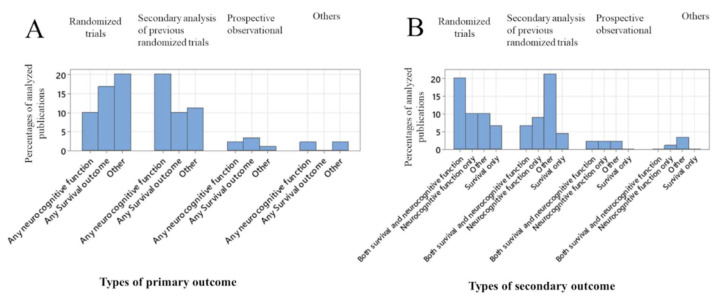
Trend of primary outcome (**A**) and secondary outcome (**B**) measurements according to types of study designs.

Figure 2 depicts the types of outcome assessments according to different types of study designs. A majority of the randomized trials used either neurocognitive function or survival as their primary outcome (Figure 2A) or secondary outcome (Figure 2B). 

## 4. Discussion

In this narrative review, we have demonstrated the trends and changes in the selection of outcomes in landmark studies in adult cardiac-arrest care. Since the publication of the COSCA initiative in 2018, we have observed an increasing trend of studies adopting outcome measures as recommended by the COSCA initiative [2]. Although our observation demonstrates a trend toward the adoption of the recommendations by Haywood et al. [2], up to 30% of our included studies still opted for other outcomes of interest. We hope that this narrative review will highlight the importance of clinical outcomes beyond survival and encourage the incorporation of higher-level outcomes in future studies. 

ROSC has long been the outcome of interest, but there are several concerns with the selection of ROSC as an outcome. First, multiple studies have shown that improved ROSC rates may not be associated with a more meaningful improvement in more-distant outcomes such as neurocognitive function or even survival [11]. In fact, some studies have shown that rates of improved ROSC may even be associated with worse neurologic outcomes [12]. Given the increasing evidence from surveys of the general population and patients indicating a strong preference for functional outcomes rather than ROSC [13,14,15,16], which may not necessarily even translate to improved rates of survival to admission (a brief episode of ROSC may still be considered a “positive” result in a study), it is imperative for higher-impact studies to avoid the use of ROSC as an outcome.

A higher-level outcome is survival to hospital admission. However, it is often argued that admission to hospital does not translate into discharge from the hospital (e.g., patient will be admitted to intensive care but die shortly after), and this is not a patient-oriented outcome; therefore, we urge that caution should be exercised in interpreting these results. Cardiac arrest is often a sudden-onset disease, unanticipated by the patient, family, and friends. It creates immense emotional distress for the family and can lead to lasting psychological harm [16]. Survival to hospital admission may allow family and friends time to process this life-altering event and provide much-needed closure. As an added benefit, it may improve the chances of organ donations to help other patients in need [17]. Nevertheless, its utility as a primary outcome is questionable.

Survival to hospital discharge, another higher-level outcome, is historically considered a superior choice, although survival to hospital discharge may not translate to good neurologic outcomes in the survivors [18], as more than 50% of discharged patients would have very poor neurologic function, and approximately 24% of cardiac-arrest survivors rely heavily on constant care [19], which, according to surveys, is not a desired outcome by many [20]. Survivors with the ability to communicate their wishes may be able to later express this to their clinicians and families; however, in the absence of the ability to clearly state their wishes, this may create both ethical and psychological dilemmas. 

This development has led to a recent shift to an even higher order of outcome that is preferred for large-scale, multicenter, and often multinational studies that are designed to inform practice guidelines. These outcomes are measured in standardized forms and include the cerebral performance category (CPC) and the modified Rankin scale (mRS). These measurements allow for a fairly reliable differentiation of the functional neurologic outcomes in survivors of cardiac arrest and for interrater reliability in the mRS or CPC [2]. As evidenced by our focused review, since the publication of the COSCA initiative, many large, multicenter, randomized controlled trials have adopted such neurologic function measurements as outcomes. However, there is a variation even when neurologic outcomes are reported. Survival to hospital discharge with a good neurologic outcome, being defined as a cerebral performance category (CPC 1–2), was reported in some studies, but there is a lack of consensus on the timeline for assessing an improvement in neurologic outcome. Although we excluded meta-analyses in this review, trial sequential analyses have been incorporated promisingly in evaluating neurological outcomes among existing studies [21,22]. In this analysis, the neurologic outcomes based on CPC or the mRS at the time of discharge or on day 28 after arrest was assessed as a secondary outcome in eight studies, respectively. We only identified two studies that assessed CPC and survival beyond 30 days. Additionally, it must be noted that neurologic function scores do not completely capture the full spectrum of cognition and psychological well-being of the survivors [2]. Even among OHCA survivors with a perceived good cognitive outcome (CPC ≤ 2), a high proportion of survivors have reduced-memory-retrieval deficits and cognitive impairment six months after arrest [23]. As such, members from the COSCA initiative, while recommending against the use of CPC in cardiac-arrest survivors, unanimously recommended mRS as the choice of neurological assessment. However, a majority of studies identified in our review still used the CPC scale as either their primary or secondary outcomes.

Due to the shortcoming of the neurologic outcome assessments, more sophisticated questionnaires such as the Health Utilities Index, the Short-Form 36-Item Health Survey, and the EuroQol 5D-5L were proposed to provide a more holistic view of the survivor’s health. Few studies are able to evaluate functional outcomes or survival along a longitudinal timeline. Up to 55% of survivors have poor functional outcome at 6 months, defined as a score of 4 to 6 on the modified Rankin scale [24]. Even among those survivors, the health-related quality-of-life score was ranked at a moderate level of 74–75 based on the EuroQol group’s visual analogue scale, with the reference range of scores of 0—“the worst health you can imagine” to 100—“the best health you can imagine” [25]. While these tools provide valuable insight into the long-term outcomes of cardiac-arrest care, they may not be optimal outcomes for interventions that are aimed at short-term outcome measurements and should not be selected as the primary outcome in such studies. Nonetheless, it is noteworthy that none of the studies in our analysis used any measure for quality of life as their outcome assessment.

It also points to the importance of an interconnected health system to capture and evaluate patients for longitudinal outcomes. Another advantage of an interconnected system is that it allows the evaluation of associated health care costs and resource utilization assessment [25]. Post-cardiac-arrest hospitalizations resulted in a high associated health care cost, with an increased length of stay, medical procedures, and systems of care [26]. The cost effectiveness of interventions should be discussed, but few studies were able to evaluate the economic impact of cardiac arrest in secondary analyses or outcome data [25,27,28].

Our review does have many limitations. First of all, it is possible that many of the trials were designed and implemented long before the publication of COSCA; thus, the authors might not have been able to change their studies’ outcomes according to the recommendation. Furthermore, we did not assess publications before the publication of the COSCA initiative to ascertain a trend in these outcome metrics before and after COSCA initiative’s recommendation. We also searched only on the PubMed database and acknowledge that we could have missed relevant studies listed on other databases. Additionally, we only included studies that reported at least one patient-related outcome; therefore, it is likely that we have artificially increased the rates of patient-related outcomes in our analysis by eliminating a large number of studies that investigated non-patient-related outcomes such as biomarker levels and quality of chest compression. 

## 5. Conclusions

Our analysis observed a trend toward an increasing number of studies using neurocognitive assessment as outcomes among the cardiopulmonary resuscitation publications since 2019. There was also a decreasing trend for the use of survival as the only outcome metric among these studies. Further studies in the field of cardiopulmonary resuscitation are necessary to confirm these trends in compliance with the recommendation by the COSCA trial.

## Data Availability

Not applicable.

## Appendix A

**Table A1:** Characteristics of studies included in the analysis.

First Author Name	Month, Year of Publication	Type of Study	Country of Study	Multisite Study	Total Patients	Name of Intervention	Name of Primary Outcome	Primary Outcome	Secondary Outcome
Akin et al.	January 2021	Prospective observational	Europe	Single-center study	251	Neuromarker analysis [29]	30 day mortality	Any survival outcome	Neurocognitive function only
Ameloot et al.	June 2019	Secondary analysis of previous randomized trial	Europe	Multicenter study	112	Mean arterial BP (MAP 65 mmHg target vs. EGDHO) [30]	Extent of anoxic brain damage (ADC reading)	Any neurocognitive function	Both survival and neurocognitive function
Ameloot et al.	August 2021	Randomized trial	Europe	Multicenter study	120	High MAP (higher dose of norepinephrine/dobutamine) [31]	Myocardial injury (area under 72 h hs-cTnT curve)	Other	Both survival and neurocognitive function
Andersen et al.	October 2021	Randomized trial	Europe	Multicenter study	501	Vasopressin and methylprednisolone [32]	Return of spontaneous circulation	Other	Both survival and neurocognitive function
Azeli et al.	May 2021	Randomized trial	Europe	Multicenter study	588	Passive leg raising (PLR) during OHCA CPR [33]	Survival to hospital discharge with good neurological outcome (CPC)	Any neurocognitive function	Both survival and neurocognitive function
Baekgaard et al.	September 2020	Secondary analysis of previous randomized trial	Europe	Multicenter study	409	Bag valve mask (BVM) vs. endotracheal intubation (ETI) [34]	Early-onset pneumonia	Other	Both survival and neurocognitive function
Baloglu Kaya et al.	March 2021	Randomized trial	Europe	Single-center study	75	Manual CPR vs. mechanical chest compression device (MCCD) [35]	rSO2 levels during CPR	Other	Both survival and neurocognitive function
Belohlavek et al.	February 2022	Randomized trial	Europe	Single-center study	256	Early invasive approach [36]	Survival with good CPC at 180 days	Any survival outcome	Neurocognitive function only
Benger et al.	April 2022	Randomized trial	Europe	Multicenter study	9289	Tracheal intubation vs. i-gel supraglottic airway as initial advanced airway management [27]	Modified Rankin score at discharge (or 30 days after OHCA, whichever occured earlier)	Any neurocognitive function	Survival only
Benger et al.	December 2020	Secondary analysis of previous randomized trial	Europe	Multicenter study	402	Tracheal intubation vs. i-gel supraglottic airway as initial advanced airway management [25]	Modified Rankin score at 30 days/hospital discharge	Any neurocognitive function	Neurocognitive function only
Berve et al.	January 2022	Randomized trial	Europe	Other/Unsure	210	Standard CPR vs. active compression–decompression CPR (ACD-CPR) [37]	Maximum tidal carbon dioxide partial pressure	Other	Survival only
Boileau et al.	June 2019	Secondary analysis of previous randomized trial	Europe	Multicenter study	590	Circulating miRNAs [38]	Poor neurological outcome at 6 months	Any neurocognitive function	Other
Cakmak et al.	April 2020	Randomized trial	Europe	Single-center study	76	Serum copeptin levels as a prediction for ROSC [39]	Serum copeptin levels	Other	Other
Cha et al.	December 2019	Randomized trial	Asia	Single-center study	163	Vitamin D deficiency (correlation with neurological outcome/mortality after resuscitation from SCA) [40]	CPC at 1 month OHCS	Any neurocognitive function	Other
Chen et al.	January 2020	Randomized trial	Europe	Single-center study	21	Plasma levels of adipokines [41]	Plasma concentrations (<1 h, 2 days, and 7 days after ROSC)	Other	Other
Cheskes et al.	May 2020	Randomized trial	Other	Multicenter study	152	Vector change defib and double sequential external defib compared to standard for pts. with VF [42]	Determine feasibility of full-scale RCT of alternative defib	Other	Other
Cheskes et al.	November 2022	Randomized trial	Other	Multicenter study	405	Vector change defib and double sequential external defib compared to standard for pts. with VF [43]	Survival to hospital discharge	Any survival outcome	Neurocognitive function only
Choi et al.	July 2022	Randomized trial	Asia	Multicenter study	150	High-/low-dose Neu2000K effect on reduction in ischemic brain injury [44]	Blood neuron-specific enolase (NSE) level on 3rd day	Other	Neurocognitive function only
Couper et al.	January 2021	Randomized trial	Europe	Multicenter study	127	Mechanical vs. manual chest compressions [45]	Proportion of eligible patients randomized during site operational recruitment	Other	Both survival and neurocognitive function
Cour et al.	May 2019	Secondary analysis of previous randomized trial	Other	Other/Unsure	33	Cyclosporine A administration [46]	Post-CA immune/ inflammatory response	Other	Other
Dankiewicz et al.	June 2021	Randomized trial	Other	Multicenter study	1850	Targeted temp management (hypothermia vs. normothermia) [24]	Death from any cause at 6 months	Any survival outcome	Neurocognitive function only
Daya et al.	January 2020	Secondary analysis of previous randomized trial	Other	Multicenter study	3019	Antiarrhythmic drugs after VF/VT (amiodarone/lidocaine/placebo) [47]	Survival to hospital discharge	Any survival outcome	Both survival and neurocognitive function
DeFazio et al.	February 2019	Secondary analysis of previous randomized trial	Europe	Multicenter study	352	Target temperature management (intravascular vs. surface cooling devices) [48]	CPC 3-5 at 6 months	Any neurocognitive function	Survival only
Desch et al.	December 2021	Randomized trial	Other/UNSURE	Multicenter study	530	Immediate vs. delayed/selective angiography [49]	Death from any cause at 30 days	Any survival outcome	Neurocognitive function only
Duez et al.	February 2019	Secondary analysis of previous randomized trial	Europe	Multicenter study	120	TTM using different EEG pattern classification models (Westhall vs. Hofmeijer) [50]	Neurological outcome using CPC after 6 months	Any neurocognitive function	Other
Düring et al.	July 2022	Secondary analysis of previous randomized trial	Other	Multicenter study	1850	TTM at 33 °C vs. normothermia [51]	All-cause mortality at 180 days	Any survival outcome	Other
Duval et al.	September 2019	Other/Unsure	Other	Multicenter study	3643	Chest compression depth and rate [52]	Optimal combination of CCR–CCD associated with functionally favorable survival	Other	Other
Eastwood et al.	July 2023	Randomized trial	Other	Multicenter study	1700	Normo vs. hypercapnia [53]	Favorable neurologic outcome (Glasgow outcome scale-extended)	Any neurocognitive function	Both survival and neurocognitive function
Ebner et al.	January 2019	Secondary analysis of previous randomized trial	Other	Multicenter study	869	Hyper vs. hypoxemia [54]	CPC at 6 months	Any neurocognitive function	Other
Elfwén et al.	June 2019	Secondary analysis of previous randomized trial	Europe	Multicenter study	79	Immediate coronary angiography [55]	Event times, procedure -related adverse events, and safety variables within 7 days	Other	Other
Evald et al.	August 2021	Secondary analysis of previous randomized trial	Europe	Multicenter study	79	Association of demography, acute care, and cerebral outcome on self-reported affective and cognitive sequelae [56]	Neuropsychological assessment	Any neurocognitive function	Neurocognitive function only
Evald et al.	January 2019	Secondary analysis of previous randomized trial	Europe	Multicenter study	79	TTM length [23]	Cognitive outcome at 6 months	Any neurocognitive function	Other
François et al.	November 2019	Randomized trial	Europe	Multicenter study	194	Amoxicillin–clavulanate antibiotic therapy (vs. saline) [57]	Early ventilator-induced pneumonia (during 1st 7 days of hospitalization)	Other	Survival only
Geri et al.	July 2019	Randomized trial	Europe	Single-center study	35	HCO-CVVHD (high-cutoff venovenous hemodialysis) [58]	Length of time between inclusion and shock resolution	Other	Other
Grand et al.	September 2019	Secondary analysis of previous randomized trial	Europe	Single-center study	151	TTM 33/36 (original study), low cardiac index (present study) [59]	180-day mortality	Any survival outcome	Other
Grand et al.	November 2020	Secondary analysis of previous randomized trial	Europe	Multicenter study	657	Mean arterial pressure [60]	Brain injury, defined at the serum level of NSE 6 months after trial	Any neurocognitive function	Neurocognitive function only
Johannes Grand	November 2020	Randomized trial	Europe	Single-center study	49	High mean arterial pressure [61]	Plasma concentration of soluble thrombomodulin (sTM) after 48 h	Other	Other
Grandfeldt et al.	June 2022	Secondary analysis of previous randomized trial	Europe	Multicenter study	501	Vasopressin and methylprednisolone [62]	6 month/1 year survival	Any survival outcome	Neurocognitive function only
Grunau et al.	December 2019	Secondary analysis of previous randomized trial	Other	Multicenter study	15,909	Epinephrine dosage [63]	Survival with favorable neurologic status at hospital discharge	Any neurocognitive function	Survival only
Grunau et al.	February 2019	Secondary analysis of previous randomized trial	Other	Multicenter study	5442	Withholding resuscitation (validation of Bokutoh criteria) [64]	Favorable neurologic outcome	Any neurocognitive function	Other
Hauw-Berlemont et al.	July 2022	Randomized trial	Europe	Multicenter study	279	Emergency coronary angiogram vs. delayed CAG for patients without ST-segment elevation [65]	180-day survival rate with CPC of 2 or less	Any neurocognitive function	Both survival and neurocognitive function
Hauw-Berlemont et al.	April 2020	Other/Unsure	Europe	Multicenter study	970	Emergency vs. delayed CAG for patients without ST-segment elevation [66]	180-day survival rate with CPC of 2 or less	Any neurocognitive function	Neurocognitive function only
Havranek et al.	December 2022	Secondary analysis of previous randomized trial	Europe	Single-center study	256	Extracorporeal cardiopulmonary (invasive) vs. standard resuscitation (role of initial rhythm) [67]	Composite 180-day survival rate with CPC 1/2	Any neurocognitive function	Survival only
Jakkula et al.	May 2019	Secondary analysis of previous randomized trial	Europe	Multicenter study	118	Cerebral oxygenation (measured with near-infrared spectroscopy) [68]	Serum NSE concentration at 48 h after CA	Other	Neurocognitive function only
Jensen et al.	February 2021	Secondary analysis of previous randomized trial	Europe	Single-center study	99	Peak systolic velocity of the mitral plane (following TTM 48 vs. 24 h) [69]	180-day neurological outcome CPC	Any neurocognitive function	Other
Johnsson et al.	July 2020	Secondary analysis of previous randomized trial	Other	Multicenter study	939	Create model for early prediction of outcome by artificial neural networks, use to examine effects on class of illness severity in CA pts treated with TTM. [70]	180-day functional outcome (CPC)	Any neurocognitive function	Other
Kander et al.	September 2019	Secondary analysis of previous randomized trial	Other	Multicenter study	722	Bleeding events after TTM [71]	Occurrence of any bleeding during the first 3 days of care	Other	Other
Kern et al.	November 2020	Randomized trial	America	Multicenter study	99	Early coronary angiography within 120 min of arrival [72]	Survival to discharge	Any survival outcome	Neurocognitive function only
Kim et al.	January 2021	Randomized trial	America	Multicenter study	1502	Amount of sodium nitrite via bolus injection [73]	Survival to hospital admission	Any survival outcome	Both survival and neurocognitive function
Kim et al.	October 2020	Prospective observational	Asia	Multicenter study	883	Ionized calcium [74]	Rate of return of spontaneous circulation	Other	Both survival and neurocognitive function
Kjaergaard et al.	October 2022	Randomized trial	Europe	Multicenter study	789	Mean arterial blood-pressure [75]	Composite mortality from any cause or hospital discharge with a good cerebral performance category score	Any survival outcome	Both survival and neurocognitive function
Lascarrou et al.	December 2019	Randomized trial	Europe	Multicenter study	584	Temperature management [76]	Survival with a favorable day-90 neurologic outcome	Any survival outcome	Neurocognitive function only
Laurikkala et al.	February 2019	Prospective observational	Europe	Multicenter study	458	Lactate measurement [77]	1-year neurologic outcome	Any neurocognitive function	Other
Le May et al.	October 2021	Randomized trial	America	Single-center study	366	Temperature management [78]	All-cause mortality or poor neurologic outcome at 180 days	Any survival outcome	Survival only
Lee et al.	February 2022	Randomized trial	Asia	Multicenter study	968	ETI and SGA insertion [79]	ROSC after cardiac arrest	Other	Both survival and neurocognitive function
Lee et al.	May 2019	Prospective observational	Asia	Multicenter study	4219	Types of shockable rhythms [80]	Survival to discharge	Any survival outcome	Neurocognitive function only
Lemkes et al.	April 2019	Randomized trial	Europe	Multicenter study	552	Coronary angiography [81]	Survial at 90 days	Any survival outcome	Neurocognitive function only
Lemkes et al.	December 2020	Secondary analysis of previous randomized trial	Europe	Multicenter study	552	Coronary angiography [82]	Survival after 1 year	Any survival outcome	Other
Lupton et al.	June 2019	Randomized trial	America	Multicenter study	2579	ETI or LT insertion [83]	Time to initial epinephrine administration from EMS arrival on scene	Other	Both survival and neurocognitive function
Lupton et al.	May 2020	Randomized trial	America	Multicenter study	3004	Airway management [84]	72 h survival after OHCA	Any survival outcome	Both survival and neurocognitive function
Moskowitz et al.	September 2020	Randomized trial	America	Multicenter study	83	Rocuronium [85]	Change in serum lactate level between enrollment and 24 h after the receipt of rucoronium	Other	Both survival and neurocognitive function
Nakashima et al.	April 2019	Prospective observational	Asia	Multicenter study	407	Targeted temperature management [86]	Favorable neurological outcome based on the CPC scale at 6 months of follow-up	Any neurocognitive function	Other
Nolan et al.	May 2020	Randomized trial	Europe	Multicenter study	7314	Adranaline or matching placebo [87]	Survival at 30 days	Any survival outcome	Both survival and neurocognitive function
Nordberg et al.	May 2019	Randomized trial	Europe	Multicenter study	671	Systemic therapeutic hypothermia [88]	Survival with good neurologic outcome 90 days after arrest	Any neurocognitive function	Survival only
Nutma et al.	May 2023	Secondary analysis of previous randomized trial	Europe	Multicenter study	157	Antiseizure medication [89]	Neurologic outcome at three months according to the cerebral performance category	Any neurocognitive function	Other
Perkins et al.	April 2021	Randomized trial	Europe	Multicenter study	8014	Adrenaline or placebo [90]	Survival to 30 days	Any survival outcome	Both survival and neurocognitive function
Prause et al.	June 2023	Randomized trial	Europe	Single-center study	46	Endotracheal intubation [91]	Adequacy of ventilation	Other	Other
Rahimi et al.	March 2022	Secondary analysis of previous randomized trial	America	Other/Unsure	1112	Amiodarone and lidocaine [92]	ROSC at hospital arrival	Other	Other
Rob et al.	October 2022	Secondary analysis of previous randomized trial	Europe	Single-center study	256	ECPR [93]	All-cause 180-day survival	Any survival outcome	Neurocognitive function only
Robba et al.	October 2022	Secondary analysis of previous randomized trial	Europe	Multicenter study	1418	Arterial blood gas value [94]	Mortality and patient neurological outcome at 6-month follow-up	Any neurocognitive function	Other
Ruijter et al.	February 2022	Randomized trial	Europe	Multicenter study	172	Antiseizure treatment [95]	Neurologic outcome at three months according to the cerebral performance category	Any neurocognitive function	Other
Schmidt et al.	October 2022	Randomized trial	Europe	Multicenter study	789	Blood pressure [96]	Composite mortality from any cause or hospital discharge with a good cerebral performance category score	Any neurocognitive function	Both survival and neurocognitive function
Skrifvars et al.	April 2020	Secondary analysis of previous randomized trial	Europe	Multicenter study	338	Temperature management [97]	Time to death until 180 days	Any survival outcome	Neurocognitive function only
Slagle et al.	February 2023	Other/Unsure	America	Single-center study	473	Hypothermia [98]	CPC	Any neurocognitive function	Other
Stokes et al.	October 2021	Other/Unsure	Europe	Other/Unsure	9296	Tracheal intubation [28]	Quality-adjusted life years (QALYs), estimated using the EQ-5D-5L questionnaire	Other	Other
Strand et al.	June 2020	Secondary analysis of previous randomized trial	Europe	Multicenter study	349	Hypothermia [99]	ICU survival	Any survival outcome	Survival only
Tissier et al.	May 2019	Secondary analysis of previous randomized trial	Europe	Multicenter study	69	Blood transcriptomics [100]	Neurological performance at day 60	Any neurocognitive function	Other
Uehara et al.	January 2023	Secondary analysis of previous randomized trial	Asia	Multicenter study	9815	ABC score [101]	Neurological outcome	Any neurocognitive function	Other
Urbano et al.	July 2022	Secondary analysis of previous randomized trial	Europe	Multicenter study	112	cEEG or rEEG [102]	Correlation between recorded EEG type and mortality	Other	Neurocognitive function only
Vallentin et al.	December 2021	Randomized trial	Europe	Multicenter study	391	Trial drug: calcium chloride [103]	Sustained return of spontaneous circulation	Other	Both survival and neurocognitive function
Vallentin et al.	July 2022	Secondary analysis of previous randomized trial	Europe	Multicenter study	391	Trial drug: calcium chloride [104]	Sustained return of spontaneous circulation	Other	Both survival and neurocognitive function
Vallentin et al.	November 2022	Randomized trial	Europe	Multicenter study	104	Trial drug: calcium chloride [105]	Return of spntaneous circulation	Other	Survival only
Vanden Berghe et al.	November 2020	Randomized trial	Europe	Multicenter study	75	Brain diffusion-weighted imaging [106]	Cerebral performance category (CPC) score at 180 days after cardiac arrest	Any neurocognitive function	Other
Wahlster et al.	June 2023	Secondary analysis of previous randomized trial	America	Multicenter study	1040	Hypothermia [107]	Incidence of early WLST-N	Other	Other
Wang et al.	December 2019	Secondary analysis of previous randomized trial	America	Multicenter study	3004	Advanced airway management with laryngeal tube or intubation [108]	Survival to 72 h after the index arrest	Any survival outcome	Both survival and neurocognitive function
Wang et al.	July 2022	Secondary analysis of previous randomized trial	America	Multicenter study	1010	Initial airway management [109]	The ventilation rate delivered a) after advanced airway insertion, and b) during airway management	Other	Both survival and neurocognitive function
Wolfrum et al.	November 2022	Randomized trial	Europe	Multicenter study	1055	Hypothermic temperature control [110]	All-cause mortality or poor neurologic outcome at 180 days	Any survival outcome	Neurocognitive function only
Yannopoulos et al.	December 2020	Randomized trial	America	Single-center study	36	Other than tandomization to 1 of 2 arms, there was no specified study intervention [111]	Survival to hospital discharge	Any survival outcome	Both survival and neurocognitive function
Yannopoulos et al.	November 2020	Prospective observational	America	Single-center study	174	Other than randomization to 1 of 2 arms, there was no specified study intervention [112]	Survival to hospital discharge	Any survival outcome	Both survival and neurocognitive function
